# Supplementary material for: Mutant RIG-I enhances cancer-related inflammation through activation of circRIG-I signaling
Source: Nat Commun. 2022 Nov 19;13:7096. doi: 10.1038/s41467-022-34885-3 (PMC9675819; doi:10.1038/s41467-022-34885-3)
Supplement: Supplementary file 1 — Supplementary Information [file 41467_2022_34885_MOESM1_ESM.pdf]

Supplementary Fig. 1

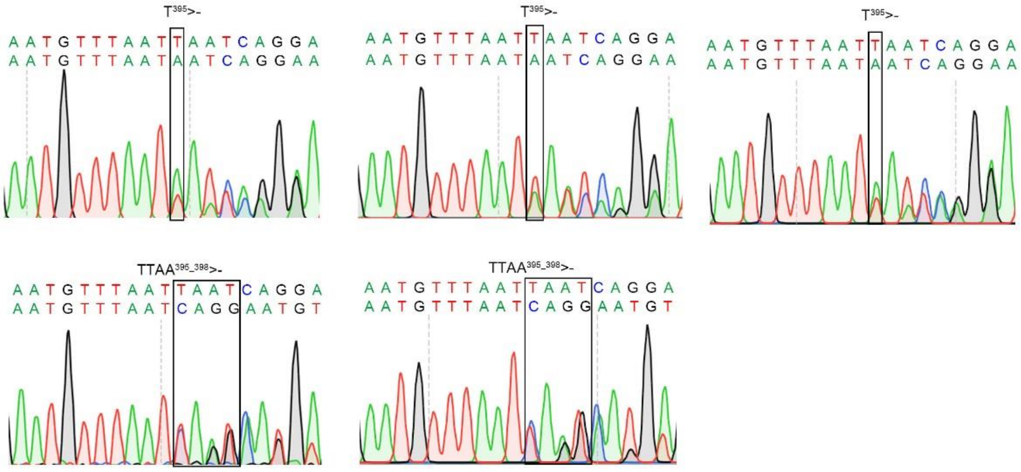

Supplementary Fig. 1. Sequence of frameshift mutation of *RIG-I* in patients with colon cancer.

The primer used for detection of *RIG-I* exon 3 has been deposited in methods and materials. Data are collected from 425 independent experiments.

Supplementary Fig. 2

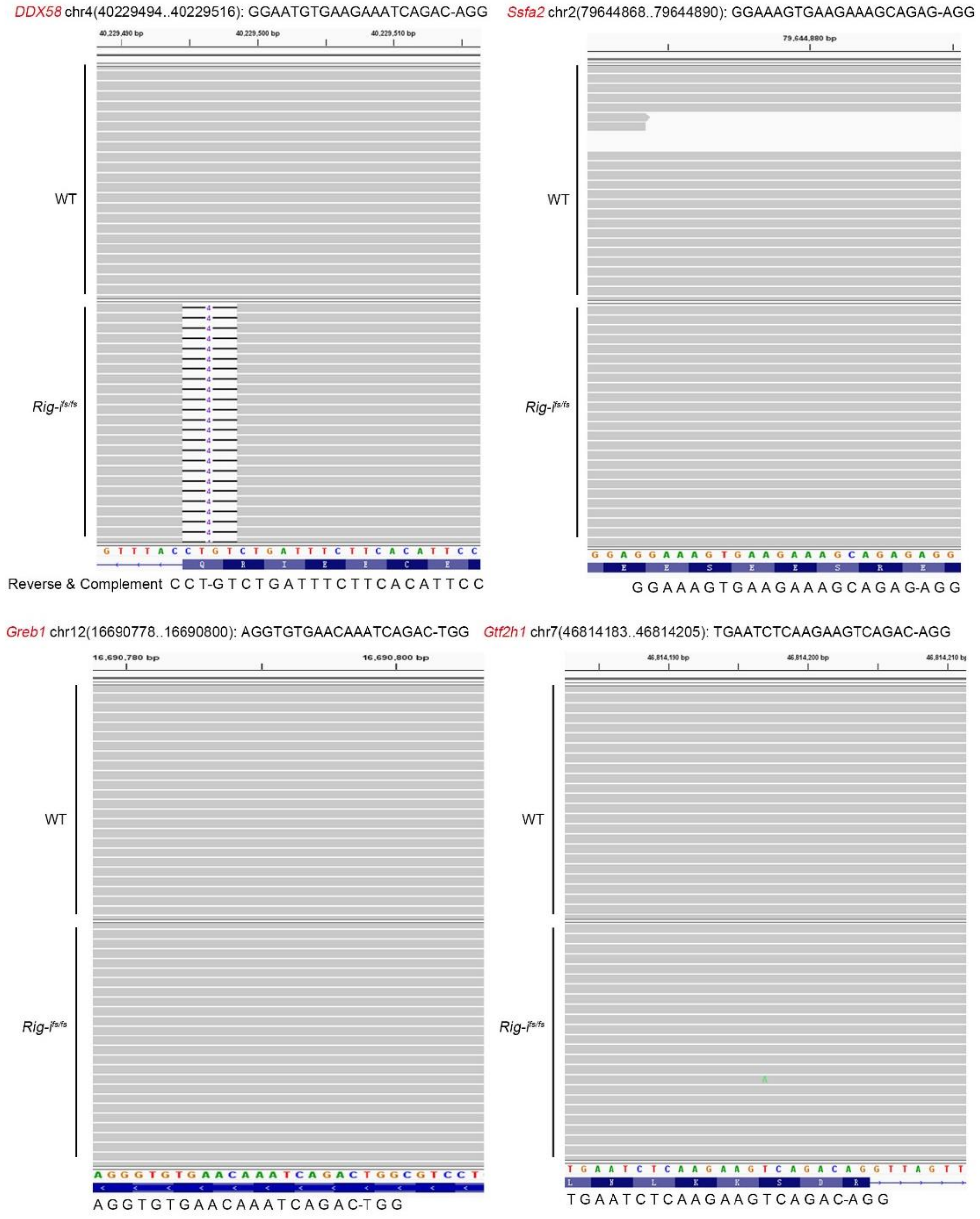

Supplementary Fig. 2. Validation of the accuracy of CRISPR-Cas9 by whole exome sequencing.

Genomic DNA was extracted from intestinal tissue of wild-type (WT) and *Rig-i*$^{fs/fs}$ mice. All potential off-targets at exons predicted by Off-Spotter (https://cm.jefferson.edu/Off-Spotter/) were examined through whole exome sequencing (WES). Neither genome mutation nor deletion of the corresponding gene was detected in *Rig-i*$^{fs/fs}$ mice and the top three ranked genes were shown. Data are collected from 2 independent experiments.

Supplementary Fig. 3

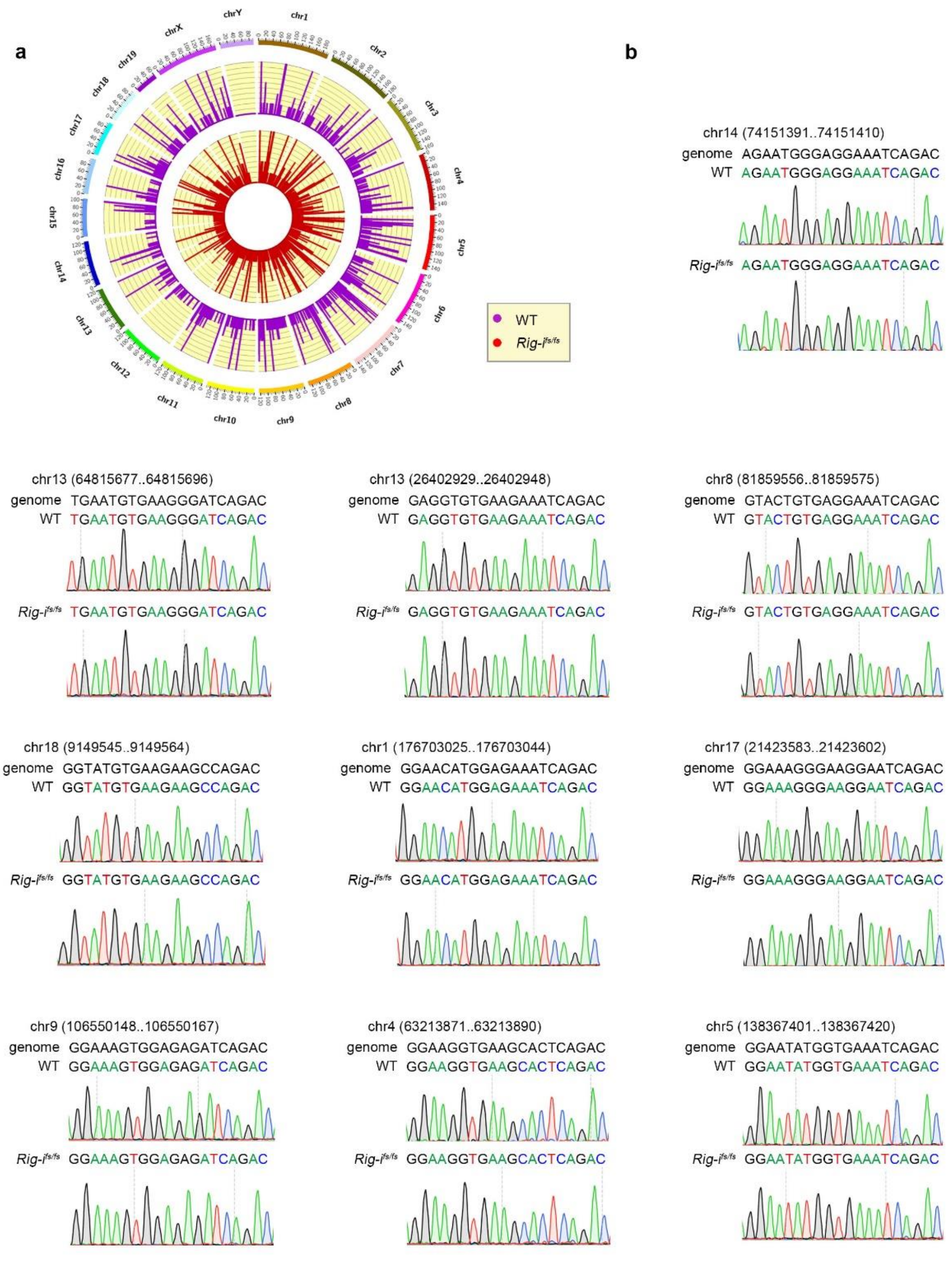

Supplementary Fig. 3. Validation of the specificity of CRISPR-Cas9 by whole exome sequencing and Sanger sequencing.

(**a**) Genomic DNA was extracted from colon tissue of wild-type and *Rig-i*$^{fs/fs}$ mice

followed by WES. Circos analysis of germline variants including single-nucleotide variant (SNV) and insertion-deletion (InDel) in wild-type (WT) and *Rig-i*$^{fs/fs}$ mice (Inner, *Rig-i*$^{fs/fs}$; Outer, WT). Data are collected from 2 independent experiments.

(**b**) Genomic DNA was extracted from intestinal tissue of wild-type and *Rig-i*$^{fs/fs}$ mice. Top ten ranked genes that may be misrecognized through Off-Spotter (https://cm.jefferson.edu/Off-Spotter/) were examined by PCR and Sanger sequencing. The primer used for analysis of potential off-targets have been deposited in **Supplementary Data 4**. Data are collected from 2 independent experiments.

Supplementary Fig. 4

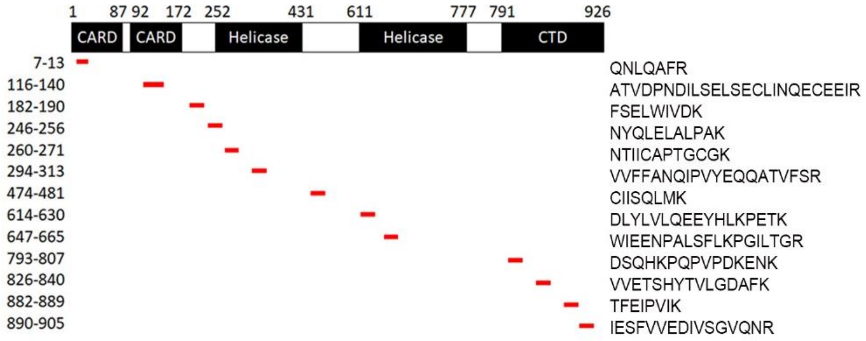

Supplementary Fig. 4. Frameshift mutation of *Rig-i* impairs its mRNA translation.

Mass spectrum analysis of RIG-I expression in wild-type and *Rig-i*$^{fs/fs}$ MEFs. Protein peptides detected in wild-type MEFs were shown. Data are collected from 2 independent experiments.

Supplementary Fig. 5

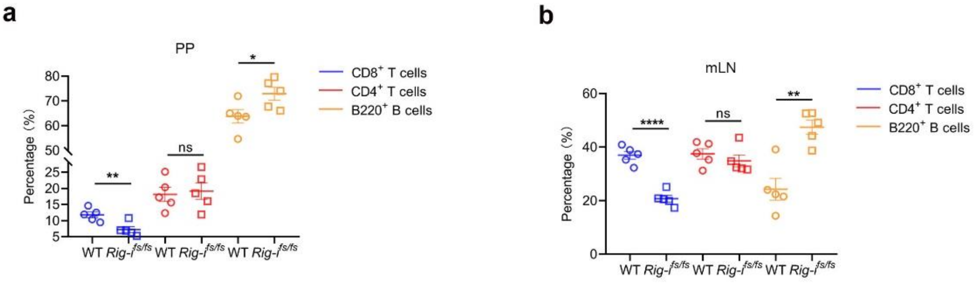

Supplementary Fig. 5. Analysis of percentages of immune cells in peripheral lymphoid organs from mice with the treatment of AOM/DSS.

(**a-b**) Flow cytometric analysis of frequency of $CD4^+$ T cell, $CD8^+$ T cell and B cell subsets in Peyer's patches (PP) (**a**) and mesenteric lymph node (mLN) (**b**) isolated from wild-type (WT) and *Rig-i*$^{fs/fs}$ mice treated with AOM/DSS (n = 5 mice, mean ± s.e.m., ns, not significant ($P > 0.05$), *$P = 0.0443$, **$P = 0.0082$ (PP), **$P = 0.0014$ (mLN), ****$P < 0.0001$, two-tailed unpaired Student’s t-test).

Source data are provided as a Source Data file.

Supplementary Fig. 6

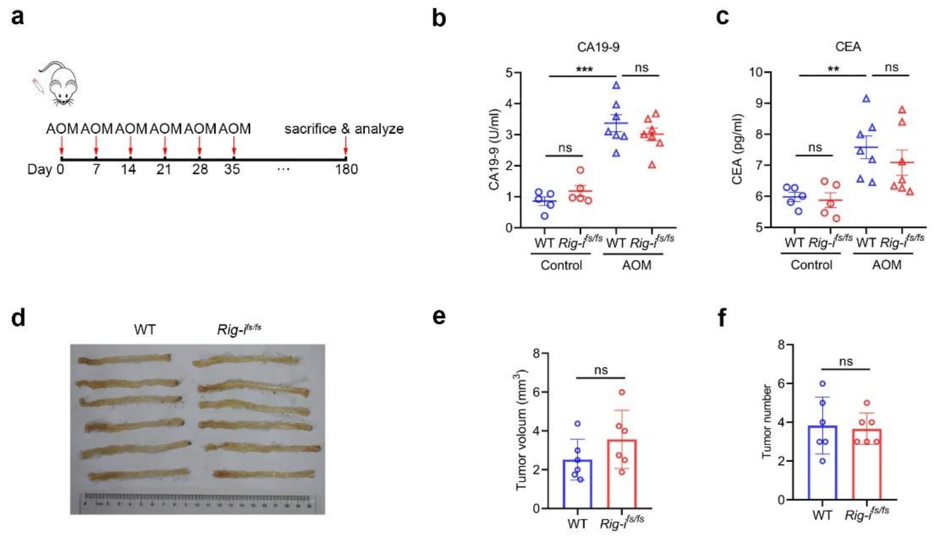

Supplementary Fig. 6. *Rig-i$^{fs/fs}$* mice exhibits same susceptibility to AOM-induced colon cancer.

(**a**) A graphic model of AOM alone assay. Wild-type (WT) and *Rig-i$^{fs/fs}$* mice were injected intraperitoneally with 10 mg/kg of AOM on day 0, day 7, day 14, day 21, day 28 and day 35. Mice were euthanized on day 180.

(**b-c**) The level of CA19-9 (**b**) and CEA (**c**) in wild-type (WT) and *Rig-i$^{fs/fs}$* mice serum was measured at day 180 in AOM-induced colon cancer model (Control group, n = 5 mice; AOM group, n = 7 mice; mean ± s.e.m., ns, not significant ($P > 0.05$), $^{**}P = 0.0054$, $^{***}P < 0.0001$, two-tailed unpaired Student's t-test).

(**d**) Representative pictures of colon tumors from wild-type (WT) and *Rig-i$^{fs/fs}$* mice in AOM-induced colon cancer model.

(**e-f**) The tumor load (**e**) or tumor number (**f**) in the whole colon was measured (n = 6 mice, mean ±s.e.m., ns, not significant ($P > 0.05$), two-tailed unpaired Student's t-test). Source data are provided as a Source Data file.

Supplementary Fig. 7

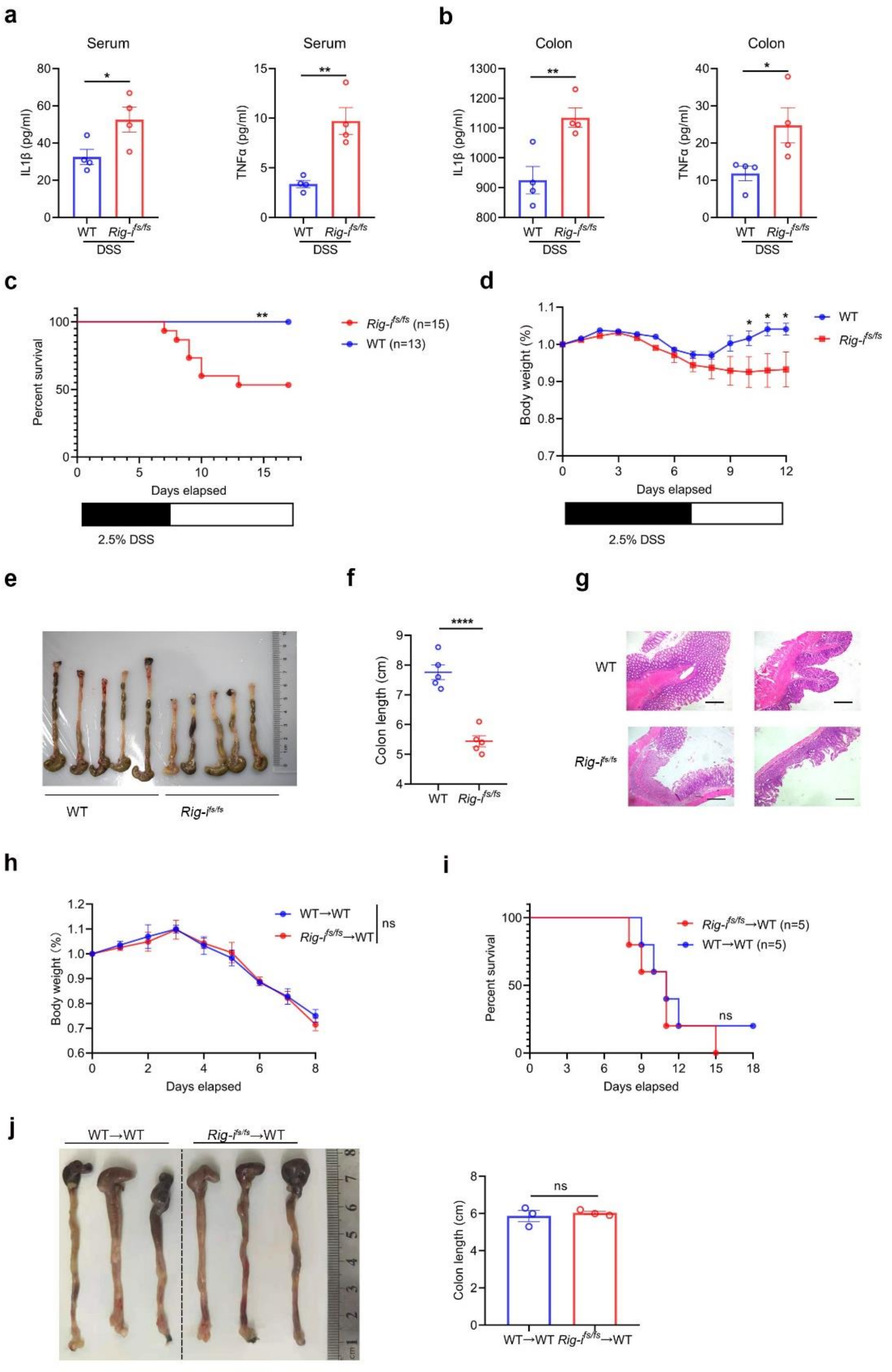

Supplementary Fig. 7. Loss of RIG-I in non-immune cell exacerbates experimental colitis.

(**a-b**) The protein levels of IL1β and TNFα in serum (n = 4 mice, mean ± s.e.m., *P = 0.0434, **P = 0.00388, two-tailed unpaired Student's t-test) (**a**) or colonic interstitial

fluid (n = 4 mice, mean ± s.e.m., *P = 0.0450, **P = 0.00989, two-tailed unpaired Student's t-test) (**b**) from wild-type and *Rig-i*$^{fs/fs}$ mice after DSS treatment were assessed by ELISA assay.

(**c**) Survival analysis of described mice with 2.5 % (weight/volume) DSS for 7 days and normal water (wild-type (WT), n=15 mice; *Rig-i*$^{fs/fs}$, n=13 mice; **P = 0.0054, Log-rank (Mantel-Cox) test).

(**d-f**) Body weight (wild-type (WT), n=13 mice; *Rig-i*$^{fs/fs}$, n=9 mice) (**d**), macroscopic evaluation (**e**) and colon length (n = 5 mice) (**f**) of wild-type (WT) and *Rig-i*$^{fs/fs}$ mice treated with 2.5 % (weight/volume) DSS for 7 days and then subjected to normal water (mean ± s.e.m., *P = 0.0422 (day 10), *P = 0.0171 (day 11), *P = 0.0214 (day 12), ****P = 0.0000706, two-tailed unpaired Student's t-test).

(**g**) Representative H&E staining pictures of colon tissues from wild-type (WT) and *Rig-i*$^{fs/fs}$ mice treated with 2.5 % (weight/volume) DSS for 7 days and then subjected to normal water. The scale bars represent 500 μm. Data are collected from 2 independent experiments.

(h-j) Bone marrow from wild-type (WT) and *Rig-i*$^{fs/fs}$ mice were transplanted into wild-type mice with irradiated (1000 cGy/mouse), respectively. 30 days after transplantation, mice were treated with DSS and assessed by body weight (n = 5 mice, mean ± s.e.m., ns, not significant (P > 0.05), two-tailed unpaired Student's t-test) (**h**), survival time (n = 5 mice, ns, not significant (P > 0.05), Log-rank (Mantel-Cox) test) (**i**) and colon length (n = 3 mice, mean ± s.e.m., ns, not significant (P > 0.05), two-tailed unpaired Student's t-test) (**j**). Source data are provided as a Source Data file.

Supplementary Fig. 8

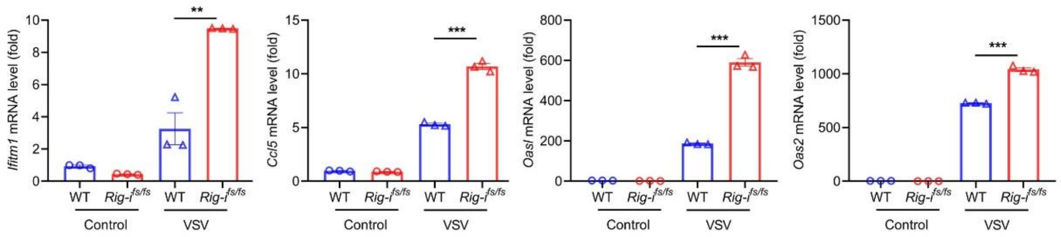

Supplementary Fig. 8. Transcriptional level of ISGs in wild-type (WT) and *Rig-i*$^{fs/fs}$ MEFs with VSV infection.

RT-qPCR analysis of mRNA levels of *Ifnβ* and *ISGs* in wild-type (WT) and *Rig-i*$^{fs/fs}$ MEFs with treatment of VSV (n = 3 cell cultures, mean ± s.e.m., ***P* = 0.0032, *** *P* < 0.0001, two-tailed unpaired Student's t-test). The primers used for quantitative real-time PCR have been deposited in **Supplementary Data 5**. Source data are provided as a Source Data file.

Supplementary Fig. 9

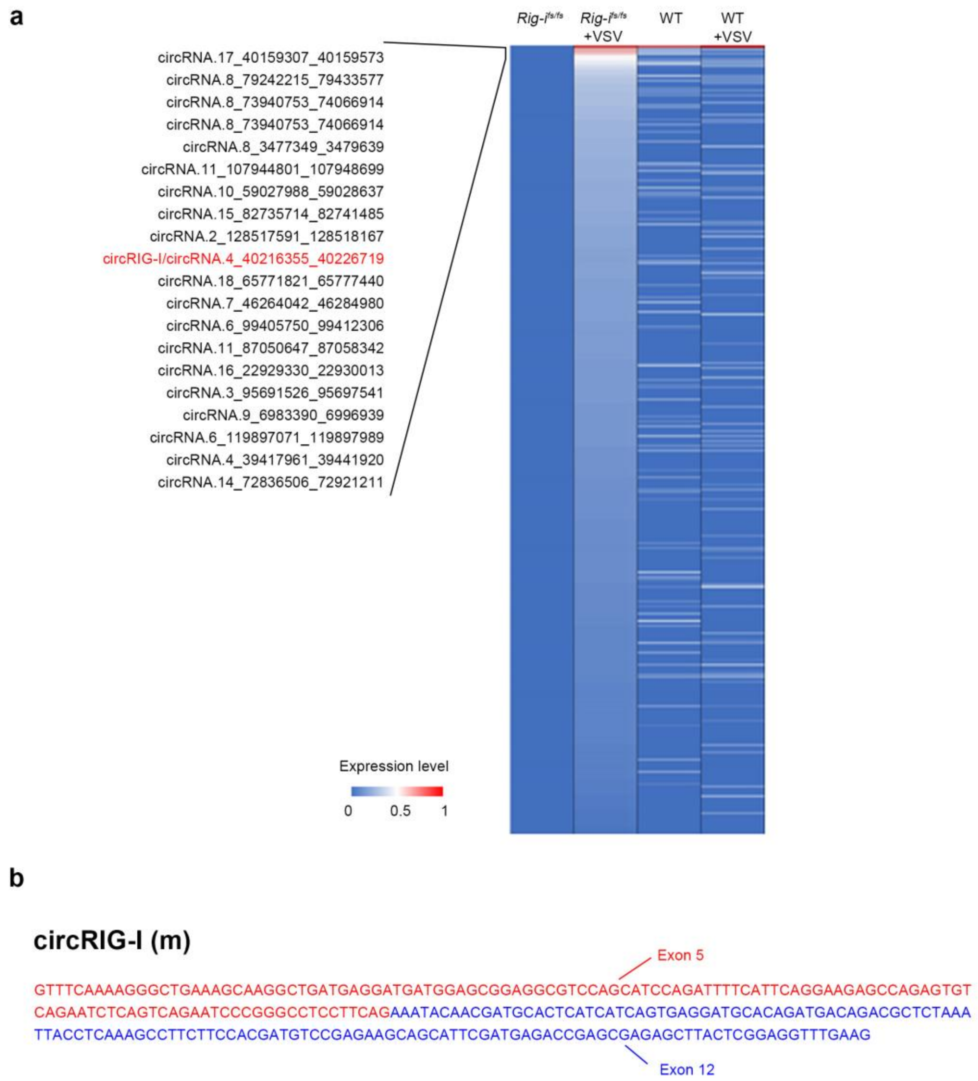

Supplementary Fig. 9. Identification of circRIG-I.

(**a**) Heatmap of circular RNA expression detected by circRNA-seq. The top 20 circRNAs upregulated in *Rig-i*$^{fs/fs}$ MEFs with VSV infection compared with *Rig-i*$^{fs/fs}$ MEFs were shown. The circRNA ID was represented by its genomic location. CircRNA.4_40216355_40226719 highlighted in red was circRIG-I in this paper.

(**b**) Sequence analysis of murine derived circRIG-I. CircRIG-I (m) was derived from exon 5 (Red) and exon 12 (Blue) of *Rig-i* gene.

Supplementary Fig. 10

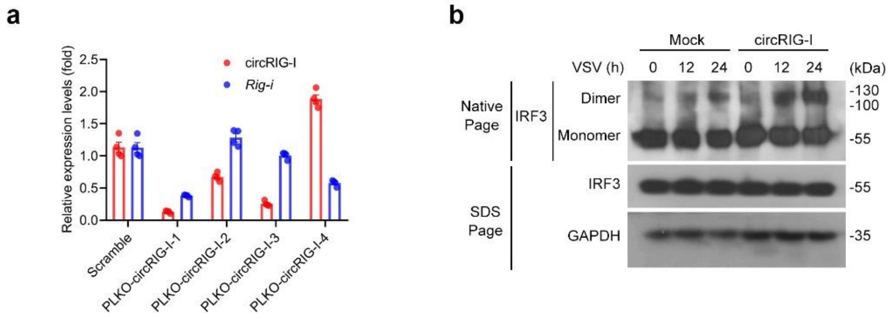

Supplementary Fig. 10. CircRIG-I promotes IRF3 dimerization and type I interferon production.

(**a**) RT-qPCR analysis of *Ddx58* mRNA level and circRIG-I level in MEFs with transfection of shRNA targeting circRIG-I (n = 4 cell cultures, mean ± s.e.m.). The primers used for quantitative real-time PCR have been deposited in **Supplementary Data 5** and targeted sequence by shRNA was detailed in methods and materials.

(**b**) Effect of circRIG-I on IRF3 dimerization. Mock or circRIG-I expressing iBMDM were treated by VSV. Followed the infection, immunoblot analysis of IRF3 in dimer or monomer form was performed by native PAGE. Data are collected from 2 independent experiments. Source data are provided as a Source Data file.

Supplementary Fig. 11

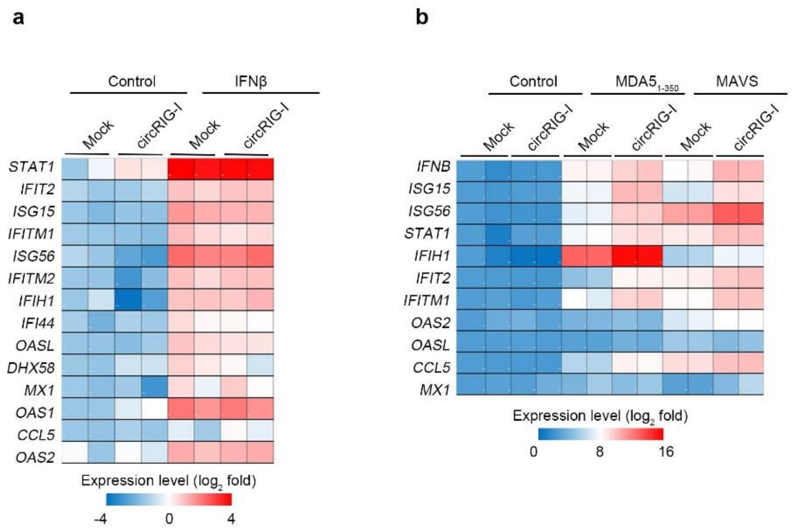

Supplementary Fig. 11. Enforced expression of CircRIG-I activates RLR signaling. (**a-b**) The mRNA levels of *IFNB* and *ISGs* in presence or absence of circRIG-I were detected by RT-qPCR (n = 2 cell cultures). The primers used for quantitative real-time PCR have been deposited in **Supplementary Data 5.** Source data are provided as a Source Data file.

Supplementary Fig. 12

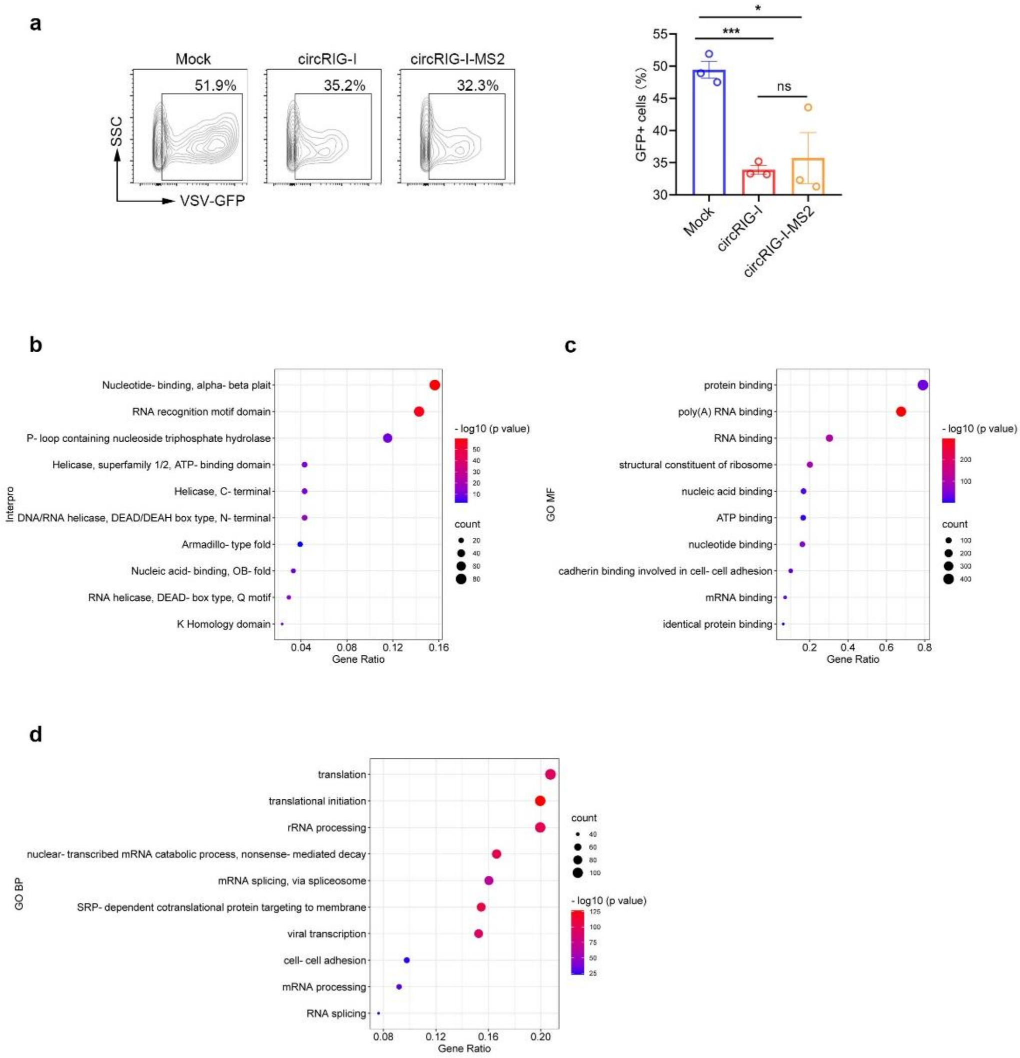

Supplementary Fig. 12. Analysis of the interactome of CircRIG-I.

(**a**) Flow cytometric analysis of GFP$^+$ HEK293T cells transfected with circRIG-I or MS2 tagged circRIG-I MEFs followed infection with VSV-GFP (n = 3 cell cultures, mean ± s.e.m., ns, not significant (P > 0.05), *P = 0.0299, ***P = 0.0004, two-tailed unpaired Student’s t-test).

(**b-d**) Enrichment analysis of the interactome of circRIG-I vs. control by Interpro and GO databases (Fisher's Exact test, one-sided，adjustments were not made for multiple comparisons.).

Source data are provided as a Source Data file.

Supplementary Fig. 13

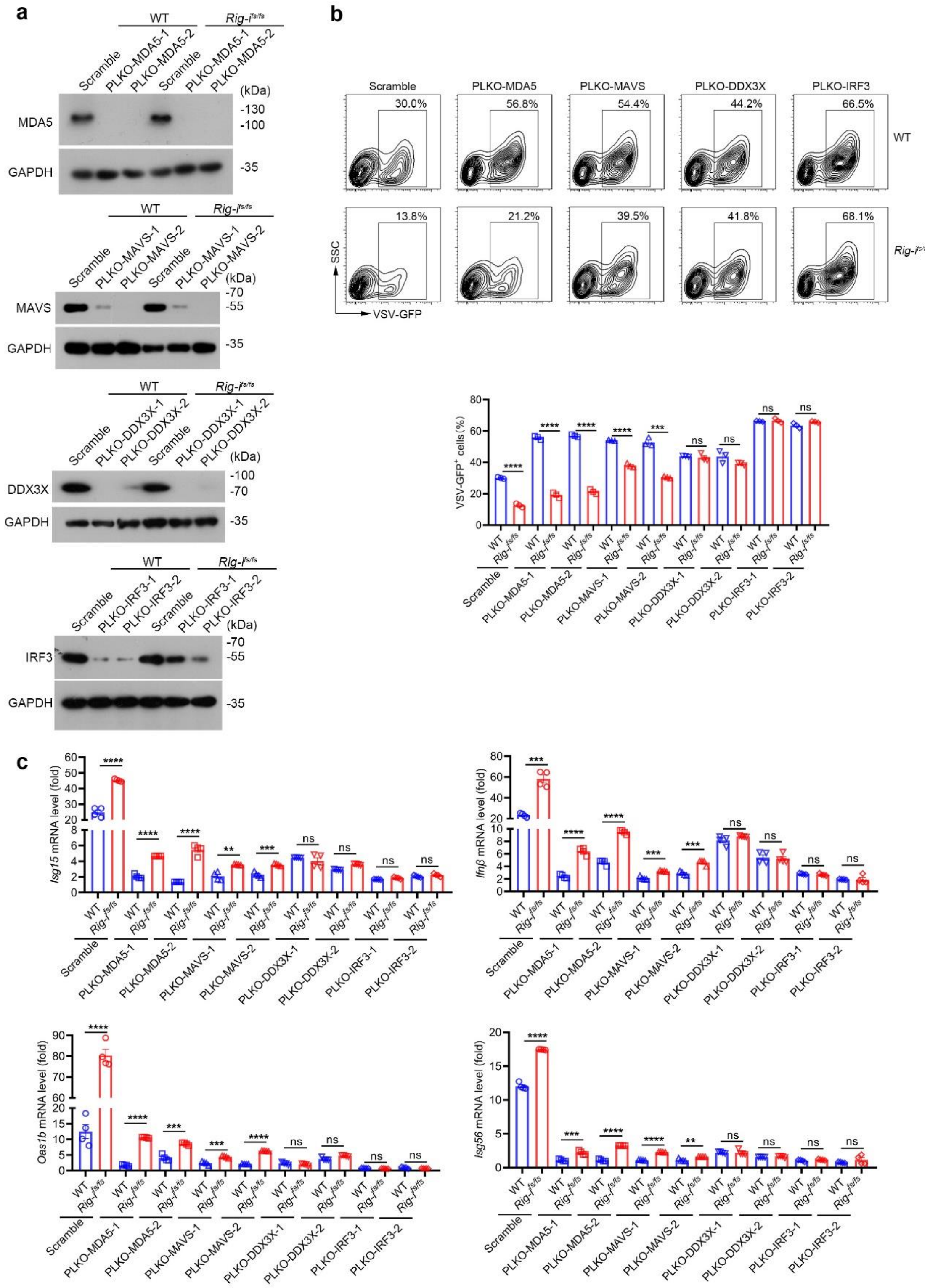

Supplementary Fig. 13. DDX3X and IRF3 are required for the antiviral effects of frameshift mutation of *Rig-i*.

(**a**) Immunoblot confirmation of the effectiveness of shRNA against *MDA5*, *MAVS*,

IRF3 as well as *DDX3X* in both wild-type and *Rig-i*$^{fs/fs}$ MEFs, respectively. Data are collected from 2 independent experiments.

(**b**) Flow cytometric analysis of GFP^{+} wild-type (WT) and *Rig-i*$^{fs/fs}$ MEFs when endogenous MDA5, MAVS, IRF3 and DDX3X were knocked down with the treatment of VSV-GFP, respectively (n = 3 cell cultures, mean ± s.e.m., ns, not significant (P > 0.05), ***P = 0.000148, **** P < 0.0001, two-tailed unpaired Student's t-test).

(**c**) RT-qPCR analysis of the mRNA levels of *Ifnβ* and other ISGs in wild-type (WT) and *Rig-i*$^{fs/fs}$ MEFs when endogenous *MDA5*, *MAVS*, *IRF3* and *DDX3X* were knocked down following VSV infection (n = 4 cell cultures, mean ± s.e.m., ns, not significant (P > 0.05), **P = 0.0013 (*Isg15*, PLKO-MAVS-1), **P = 0.0019 (*Isg56*, PLKO-MAVS-2), ***P = 0.0005 (*Isg15*, PLKO-MAVS-1), ***P = 0.0001 (*Ifnβ*, Scamble), ***P = 0.0002 (*Isg56*, PLKO-MDA5-1), ***P = 0.0005 (*Ifnβ*, PLKO-MAVS-1), ***P = 0.0002 (*Ifnβ*, PLKO-MAVS-2), ***P = 0.0009 (*Oas1b*, PLKO-MDA5-2), ***P = 0.0008 (*Oas1b*, PLKO-MAVS-1), **** P < 0.0001, two-tailed unpaired Student's t-test).

Source data are provided as a Source Data file.

Supplementary Fig. 14

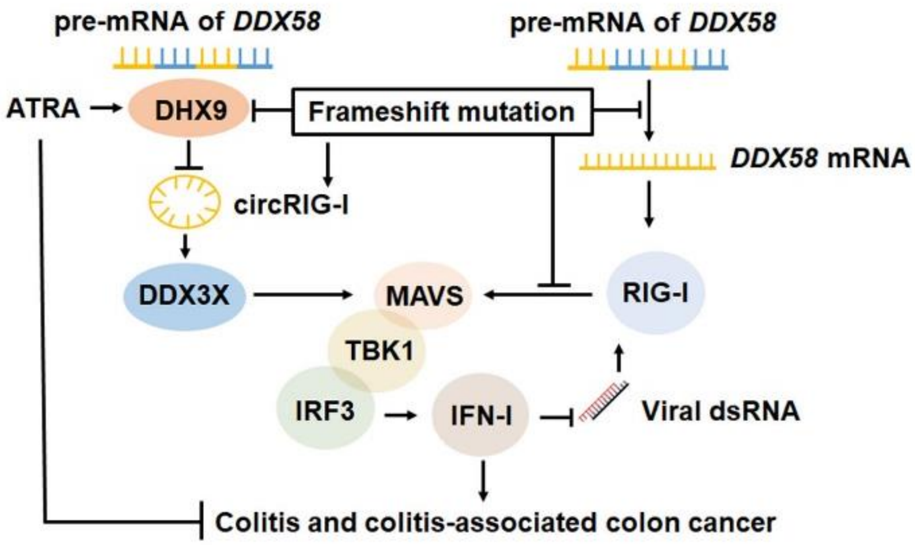

Supplementary Fig. 14. Model for the role of circRIG-I in host antitumor immunity.

Frameshift mutation of *RIG-I* triggers circRIG-I generation and enhances host innate immune response by activation of DDX3X/MAVS/TRAF5/TBK1 pathway. CircRIG-Is increases susceptibility to colitis and colitis-associated cancer, which can be reversed by ATRA treatment.

Supplementary Fig. 15

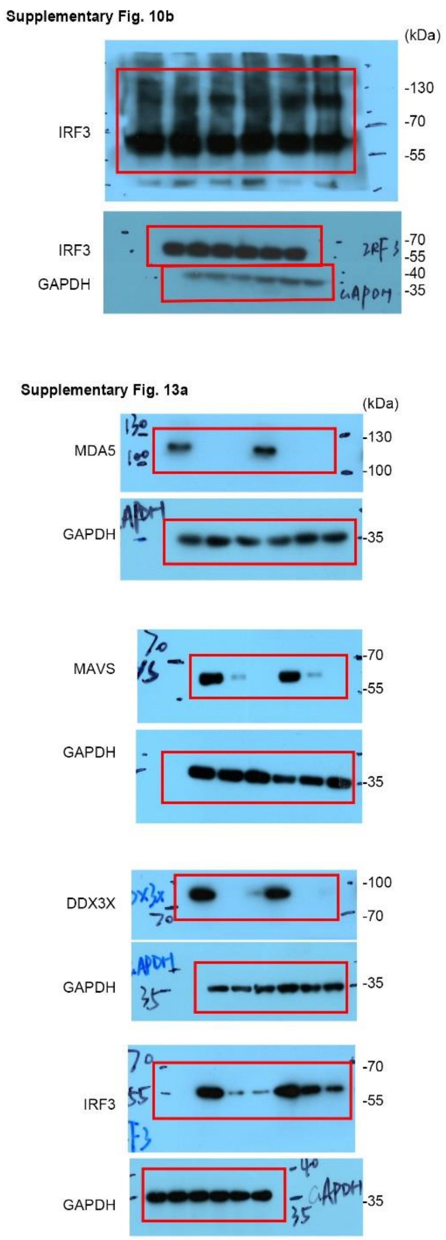

Supplementary Figure 15. Uncropped scans of blots in Supplementary Figures.
