# Supplementary material for: Mutant RIG-I enhances cancer-related inflammation through activation of circRIG-I signaling
Source: Nat Commun. 2022 Nov 19;13:7096. doi: 10.1038/s41467-022-34885-3 (PMC9675819; doi:10.1038/s41467-022-34885-3)
Supplement: Supplementary file 3 — Description of Additional Supplementary Files [file 41467_2022_34885_MOESM3_ESM.pdf]

Description of Additional Supplementary Files:

Supplementary Data 1: Clinical information of colon cancer patients.

Supplementary Data 2: CircRIG-I associated proteins by proximity-based labeling coupled mass cytometry.

Supplementary Data 3: DDX3X associated proteins in the presence or absence of circRIG-I.

Supplementary Data 4: The primers used for detection of potential off-targets.

Supplementary Data 5: The primers used for quantitative real-time PCR.
