# Supplementary material for: Mutant RIG-I enhances cancer-related inflammation through activation of circRIG-I signaling
Source: Nat Commun. 2022 Nov 19;13:7096. doi: 10.1038/s41467-022-34885-3 (PMC9675819; doi:10.1038/s41467-022-34885-3)
Supplement: Supplementary file 9 — Reporting Summary [file 41467_2022_34885_MOESM9_ESM.pdf]

nature portfolio

Corresponding author(s): Dan Lu
Last updated by author(s): Nov 4, 2022

Reporting Summary

Nature Portfolio wishes to improve the reproducibility of the work that we publish. This form provides structure for consistency and transparency in reporting. For further information on Nature Portfolio policies, see our Editorial Policies and the Editorial Policy Checklist.

Statistics

For all statistical analyses, confirm that the following items are present in the figure legend, table legend, main text, or Methods section.

n/a	Confirmed	
☐	☒	The exact sample size (n) for each experimental group/condition, given as a discrete number and unit of measurement
☐	☒	A statement on whether measurements were taken from distinct samples or whether the same sample was measured repeatedly
☐	☒	The statistical test(s) used AND whether they are one- or two-sided<br>*Only common tests should be described solely by name; describe more complex techniques in the Methods section.*
☒	☐	A description of all covariates tested
☐	☒	A description of any assumptions or corrections, such as tests of normality and adjustment for multiple comparisons
☐	☒	A full description of the statistical parameters including central tendency (e.g. means) or other basic estimates (e.g. regression coefficient) AND variation (e.g. standard deviation) or associated estimates of uncertainty (e.g. confidence intervals)
☐	☒	For null hypothesis testing, the test statistic (e.g. F, t, r) with confidence intervals, effect sizes, degrees of freedom and P value noted<br>*Give P values as exact values whenever suitable.*
☒	☐	For Bayesian analysis, information on the choice of priors and Markov chain Monte Carlo settings
☒	☐	For hierarchical and complex designs, identification of the appropriate level for tests and full reporting of outcomes
☒	☐	Estimates of effect sizes (e.g. Cohen's d, Pearson's r), indicating how they were calculated

Our web collection on statistics for biologists contains articles on many of the points above.

Software and code

Policy information about availability of computer code

Data collection	Flow cytometry: FACSuite Software Bundle v1.0 (BD Biosciences).<br>Image of Con-focal assay: NIS-Elements AR Analysis 4.20.00 64bit (Nikon)<br>qRT-PCR: 7500 Software v2.3 (Applied Biosystems)<br>Image of the histological staining:'cellSens'software (Olympus)
Data analysis	GraphPad Prism v7.0 was used for statistical analyses.<br>NIS Elements Viewer 4.20 was used for confocal images.<br>FlowJo v7.6.1 was used to analyze the flow cytometry data.<br>Clean reads in RNA-seq were mapped with the reference genome Hisat2 (version 2.0.5) based on the gene model annotation file<br>Feature Counts v1.5.0-p3 was used to count the reads in the RNA-seq data.<br>GSEA 3.0 analysis tool (http://software.broadinstitute.org/gsea/index.jsp) was used for signaling analysis.

For manuscripts utilizing custom algorithms or software that are central to the research but not yet described in published literature, software must be made available to editors and reviewers. We strongly encourage code deposition in a community repository (e.g. GitHub). See the Nature Portfolio guidelines for submitting code & software for further information.

Data

Policy information about availability of data

All manuscripts must include a data availability statement. This statement should provide the following information, where applicable:

- Accession codes, unique identifiers, or web links for publicly available datasets
- A description of any restrictions on data availability
- For clinical datasets or third party data, please ensure that the statement adheres to our policy

The RNA-seq data generated in this study have been deposited in the SRA database under accession codes PRJNA741380 [https://www.ncbi.nlm.nih.gov/bioproject/?term=PRJNA741380] and PRJNA759839 [https://www.ncbi.nlm.nih.gov/bioproject/PRJNA759839]. The circRNA-seq data generated in this study have been deposited in the SRA database under accession code PRJNA782066 [https://www.ncbi.nlm.nih.gov/bioproject/PRJNA782066]. The mass spectrometry proteomics data have been deposited to the ProteomeXchange Consortium via the PRIDE partner repository with the dataset identifier PXD037928 [https://www.ebi.ac.uk/pride/archive/projects/PXD037928] and PXD037929 [https://www.ebi.ac.uk/pride/archive/projects/PXD037929]. The WES data generated in this study have been deposited in the SRA database under accession code PRJNA897090 [https://www.ncbi.nlm.nih.gov/bioproject/PRJNA897090].

Field-specific reporting

Please select the one below that is the best fit for your research. If you are not sure, read the appropriate sections before making your selection.

☒ Life sciences ☐ Behavioural & social sciences ☐ Ecological, evolutionary & environmental sciences

For a reference copy of the document with all sections, see nature.com/documents/nr-reporting-summary-flat.pdf

Life sciences study design

All studies must disclose on these points even when the disclosure is negative.

Sample size	Sample size was chosen based on the standard practices in the field. Sample size was chosen based on previous experience by our group on similar experiments (PMID 26479789 and 31932812).
Data exclusions	No data were excluded from the analysis.
Replication	All experiments were independently replicated at least three times and similar results were generated.
Randomization	For animal studies using WT and Rig-ifs/fs mice, no randomization was performed, and samples were grouped by genotypes. Other animals or cells were measured at random within each condition.
Blinding	Data acquisition and analyses were not blinded but all assays were performed at the same time for all groups of a given experiment. Since all conditions were subjected to the same analyses, blinding was not considered to be necessary.

Behavioural & social sciences study design

All studies must disclose on these points even when the disclosure is negative.

Study description	*Briefly describe the study type including whether data are quantitative, qualitative, or mixed-methods (e.g. qualitative cross-sectional, quantitative experimental, mixed-methods case study).*
Research sample	*State the research sample (e.g. Harvard university undergraduates, villagers in rural India) and provide relevant demographic information (e.g. age, sex) and indicate whether the sample is representative. Provide a rationale for the study sample chosen. For studies involving existing datasets, please describe the dataset and source.*
Sampling strategy	*Describe the sampling procedure (e.g. random, snowball, stratified, convenience). Describe the statistical methods that were used to predetermine sample size OR if no sample-size calculation was performed, describe how sample sizes were chosen and provide a rationale for why these sample sizes are sufficient. For qualitative data, please indicate whether data saturation was considered, and what criteria were used to decide that no further sampling was needed.*
Data collection	*Provide details about the data collection procedure, including the instruments or devices used to record the data (e.g. pen and paper, computer, eye tracker, video or audio equipment) whether anyone was present besides the participant(s) and the researcher, and whether the researcher was blind to experimental condition and/or the study hypothesis during data collection.*
Timing	*Indicate the start and stop dates of data collection. If there is a gap between collection periods, state the dates for each sample cohort.*
Data exclusions	*If no data were excluded from the analyses, state so OR if data were excluded, provide the exact number of exclusions and the rationale behind them, indicating whether exclusion criteria were pre-established.*

Non-participation	*State how many participants dropped out/declined participation and the reason(s) given OR provide response rate OR state that no participants dropped out/declined participation.*
Randomization	*If participants were not allocated into experimental groups, state so OR describe how participants were allocated to groups, and if allocation was not random, describe how covariates were controlled.*

Ecological, evolutionary & environmental sciences study design

All studies must disclose on these points even when the disclosure is negative.

Study description	*Briefly describe the study. For quantitative data include treatment factors and interactions, design structure (e.g. factorial, nested, hierarchical), nature and number of experimental units and replicates.*
Research sample	*Describe the research sample (e.g. a group of tagged Passer domesticus, all Stenocereus thurberi within Organ Pipe Cactus National Monument), and provide a rationale for the sample choice. When relevant, describe the organism taxa, source, sex, age range and any manipulations. State what population the sample is meant to represent when applicable. For studies involving existing datasets, describe the data and its source.*
Sampling strategy	*Note the sampling procedure. Describe the statistical methods that were used to predetermine sample size OR if no sample-size calculation was performed, describe how sample sizes were chosen and provide a rationale for why these sample sizes are sufficient.*
Data collection	*Describe the data collection procedure, including who recorded the data and how.*
Timing and spatial scale	*Indicate the start and stop dates of data collection, noting the frequency and periodicity of sampling and providing a rationale for these choices. If there is a gap between collection periods, state the dates for each sample cohort. Specify the spatial scale from which the data are taken*
Data exclusions	*If no data were excluded from the analyses, state so OR if data were excluded, describe the exclusions and the rationale behind them, indicating whether exclusion criteria were pre-established.*
Reproducibility	*Describe the measures taken to verify the reproducibility of experimental findings. For each experiment, note whether any attempts to repeat the experiment failed OR state that all attempts to repeat the experiment were successful.*
Randomization	*Describe how samples/organisms/participants were allocated into groups. If allocation was not random, describe how covariates were controlled. If this is not relevant to your study, explain why.*
Blinding	*Describe the extent of blinding used during data acquisition and analysis. If blinding was not possible, describe why OR explain why blinding was not relevant to your study.*

Did the study involve field work? ☐ Yes ☐ No

Field work, collection and transport

Field conditions	*Describe the study conditions for field work, providing relevant parameters (e.g. temperature, rainfall).*
Location	*State the location of the sampling or experiment, providing relevant parameters (e.g. latitude and longitude, elevation, water depth).*
Access & import/export	*Describe the efforts you have made to access habitats and to collect and import/export your samples in a responsible manner and in compliance with local, national and international laws, noting any permits that were obtained (give the name of the issuing authority, the date of issue, and any identifying information).*
Disturbance	*Describe any disturbance caused by the study and how it was minimized.*

Reporting for specific materials, systems and methods

We require information from authors about some types of materials, experimental systems and methods used in many studies. Here, indicate whether each material, system or method listed is relevant to your study. If you are not sure if a list item applies to your research, read the appropriate section before selecting a response.

Materials & experimental systems

n/a	Involved in the study	
	☒	Antibodies
	☒	Eukaryotic cell lines
☒		Palaeontology and archaeology
	☒	Animals and other organisms
	☒	Human research participants
☒		Clinical data
☒		Dual use research of concern

Methods

n/a	Involved in the study	
☒		ChIP-seq
	☒	Flow cytometry
☒		MRI-based neuroimaging

Antibodies

Antibodies used

Antibodies used in this study were as follows, clone number, catalogue number and dilutions were shown in turn:
anti-RIG-I (Santa Cruz Biotechnology, sc-376845, 1:1000), anti-p-IRF3 (Cell Signaling Technology, #4947, 1:1000), anti-MDA5 (Abclonal, A2419, 1:500), anti-MAVS (abcam, ab189109,1:2000), anti-IRF3 (abcam, ab68481, 1:2000), anti-DDX3X (Santa Cruz Biotechnology, sc-365768, 1:500), anti-DHX9 (Santa Cruz Biotechnology, sc-137232, 1:500), anti-GAPDH (RayAntibody, RM2002, 1:5000), anti-α-tubulin (RayAntibody, RM2007, 1:5000), anti-HDAC1(Santa Cruz Biotechnology, sc-8410, 1:1000), anti-FLAG (Sigma, F3165, 1:5000), anti-GFP (RayAntibody, RM1008, 1:5000), APC-labeled anti-CD4 antibody (Biolegend, GK1.5, 1:500), FITC-labeled anti-CD8 antibody (Biolegend, 53-6.7, 1:500), PE-labeled anti-B220 antibody (Biolegend, RA3-6B2, 1:500), KPL peroxidase-labeled antibody to mouse IgG (H+L) (Seracare, 5220-0341, 1:5000) and KPL peroxidase-labeled antibody to rabbit IgG (H+L) (Seracare, 5220-0336, 1:5000).

Validation

All antibodies used in the study were bought commercially, and all commercially antibodies have been validated by the manufacturer as stated on their websites. Manufacturers state the antibodies have been validated for intended uses. Manufacturer citations are listed in manufacturer websites for each specific antibody. Websites of the manufacturers were as follows:
anti-RIG-I (Santa Cruz Biotechnology, sc-376845) https://www.scbt.com/p/rig-i-antibody-d-12?requestFrom=search
anti-p-IRF3 (Cell Signaling Technology, #4947) https://www.cellsignal.cn/products/primary-antibodies/phospho-irf-3-ser396-4d4g-rabbit-mab/4947?site-search-type=Products&N=4294956287&Ntt=4947&fromPage=plp&_requestid=6917489
anti-MDA5 (Abclonal, A2419) https://abclonal.com.cn/catalog/A2419
anti-MAVS (abcam, ab189109) https://www.abcam.cn/mavs-antibody-ab189109.html
anti-IRF3 (abcam, ab68481) https://www.abcam.cn/irf3-antibody-epr2418y-ab68481.html
anti-DDX3X (Santa Cruz Biotechnology, sc-365768) https://www.scbt.com/zh/p/ddx3-antibody-c-4?requestFrom=search
anti-DHX9 (Santa Cruz Biotechnology, sc-137232) https://www.scbt.com/zh/p/ndh-ii-antibody-b-9?requestFrom=search
anti-GAPDH (RayAntibody, RM2002) http://www.rayantibody.com/uploads/2014/07/101628307560.pdf
anti-α-tubulin (RayAntibody, RM2007) http://www.rayantibody.com/uploads/2014/07/101708444084.pdf
anti-HDAC1(Santa Cruz Biotechnology, sc-8410) https://www.scbt.com/zh/p/hdac1-antibody-h-11?requestFrom=search
anti-FLAG (Sigma, F3165) https://www.sigmaaldrich.cn/CN/zh/product/sigma/f3165
anti-GFP (RayAntibody, RM1008) http://www.rayantibody.com/uploads/2014/07/101640111509.pdf
APC-labeled anti-CD4 antibody (Biolegend, GK1.5) https://www.biolegend.com/en-us/products/apc-anti-mouse-cd4-antibody-245
FITC-labeled anti-CD8 antibody (Biolegend, 53-6.7) https://www.biolegend.com/en-us/products/fitc-anti-mouse-cd8a-antibody-153
PE-labeled anti-B220 antibody (Biolegend, RA3-6B2) https://www.biolegend.com/en-us/products/pe-anti-mouse-human-cd45r-b220-antibody-447
KPL peroxidase-labeled antibody to mouse IgG (H+L) (Seracare, 5220-0341) https://www.seracare.com/AntiMouse-IgG-HL-Antibody-Human-Serum-Adsorbed-and-PeroxidaseLabeled-5220-0341/
KPL peroxidase-labeled antibody to rabbit IgG (H+L) (Seracare, 5220-0336) https://www.seracare.com/AntiRabbit-IgG-HL-Antibody-PeroxidaseLabeled-5220-0336/

Eukaryotic cell lines

Policy information about cell lines

Cell line source(s)

HEK293T cells were obtained from American Type Culture Collection (ATCC).
Primary mouse embryonic fibroblasts (MEFs) from wild-type (WT) and Rig-ifs/fs mouse embryos at 13.5-day post-coitum.
Immortalized bone-marrow-derived macrophages (iBMDMs) were provided by Dr. Fuping You (Peking University Health Science Center, China).

Authentication

None of the cell lines were authenticated.

Mycoplasma contamination

All of the cell lines were negative for mycoplasma contamination.

Commonly misidentified lines
(See ICLAC register)

No commonly misidentified cell lines were used.

Palaeontology and Archaeology

Specimen provenance

Provide provenance information for specimens and describe permits that were obtained for the work (including the name of the issuing authority, the date of issue, and any identifying information). Permits should encompass collection and, where applicable, export.

Specimen deposition

Indicate where the specimens have been deposited to permit free access by other researchers.

Dating methods

If new dates are provided, describe how they were obtained (e.g. collection, storage, sample pretreatment and measurement), where they were obtained (i.e. lab name), the calibration program and the protocol for quality assurance OR state that no new dates are provided.

☐ Tick this box to confirm that the raw and calibrated dates are available in the paper or in Supplementary Information.

Ethics oversight

Identify the organization(s) that approved or provided guidance on the study protocol, OR state that no ethical approval or guidance was required and explain why not.

Note that full information on the approval of the study protocol must also be provided in the manuscript.

Animals and other organisms

Policy information about studies involving animals; ARRIVE guidelines recommended for reporting animal research

Laboratory animals

C57BL/6J mice were purchased from Beijing Vital River Laboratory Animal Technology Co., Ltd. All animals were housed and maintained under specific pathogen-free conditions. 6-8 weeks old male mice were used for the study. The mice were reared in standard conditions with controlled temperature (20-26℃° C), humidity (40-70%) and 12/12-hour dark/light cycle.

Wild animals

No wild animals were used.

Field-collected samples

No field-collected samples were used in the study.

Ethics oversight

All animal experiments were performed in accordance with protocols approved by the Ethics Committee of Peking University Health Science Center.

Note that full information on the approval of the study protocol must also be provided in the manuscript.

Human research participants

Policy information about studies involving human research participants

Population characteristics

Accession number Tumor classification Morphological classification Grade
CRC-1 Colon Mucinous adenocarcinoma Polypoid pT3N2a
CRC-2 Rectum Mucinous adenocarcinoma Polypoid pT3N0
CRC-3 Ileocecus Adenocarcinoma Ulcerative pT3N2a
CRC-4 Colon Adenocarcinoma Unknown pT4aN1a
CRC-5 Colon Adenocarcinoma Polypoid pT3N0
CRC-6 Colon Adenocarcinoma Unknown pT3N0
CRC-7 Rectum Adenocarcinoma Ulcerative pT2N2a
CRC-8 Colon Mucinous adenocarcinoma Ulcerative pT3N2a
CRC-9 Colon Adenocarcinoma Ulcerative pT3N0
CRC-10 Colon Adenocarcinoma Polypoid pT2N2a
CRC-11 Colon Adenocarcinoma Unknown pT1N1a
CRC-12 Rectum Adenocarcinoma Polypoid pT3N0
CRC-13 Colon Adenocarcinoma Ulcerative pT3N0
CRC-14 Rectum Adenocarcinoma Unknown pT3N0
CRC-15 Rectum Adenocarcinoma Ulcerative pT3N0
CRC-16 Rectum Mucinous adenocarcinoma Ulcerative pT3N0
CRC-17 Colon Adenocarcinoma Ulcerative pT3N0
CRC-18 Colon Adenocarcinoma Polypoid pT3N0
CRC-19 Colon Mucinous adenocarcinoma Ulcerative pT4aN1a
CRC-20 Colon Adenocarcinoma Unknown pT3N0
CRC-21 Rectum Adenocarcinoma Unknown pT3N0
CRC-22 Rectum Adenocarcinoma Polypoid PT2N0
CRC-23 Colon Adenocarcinoma Ulcerative pT3N1b
CRC-24 Rectum Adenocarcinoma Polypoid pT3N0
CRC-25 Colon Adenocarcinoma Ulcerative pT3N0
CRC-26 Colon Adenocarcinoma Polypoid pT2N1a
CRC-27 Rectum Adenocarcinoma Unknown PT1N0
CRC-28 Rectum Mucinous adenocarcinoma Polypoid pT3N1a
CRC-29 Rectum Adenocarcinoma Ulcerative pT2N1b

CRC-30	Rectum	Adenocarcinoma	Polypoid	pT3N0
CRC-31	Colon	Adenocarcinoma	Unknown	pT3N0
CRC-32	Colon	Adenocarcinoma	Ulcerative	pT3N0
CRC-33	Rectum	Adenocarcinoma	Unknown	pT4aN2b
CRC-34	Rectum	Adenocarcinoma	Ulcerative	pT3N1b
CRC-35	Colon	Mucinous adenocarcinoma	Unknown	pT3N0
CRC-36	Colon	Adenocarcinoma	Unknown	pT3N2b
CRC-37	Rectum	Adenocarcinoma	Ulcerative	pT3N2a
CRC-38	Colon	Adenocarcinoma	Unknown	pT2N0
CRC-39	Ileocecus	Adenocarcinoma	Ulcerative	pT4bN0
CRC-40	Rectum	Adenocarcinoma	Ulcerative	pT3N0
CRC-41	Colon	Adenocarcinoma	Unknown	pT3N1b
CRC-42	Rectum	Adenocarcinoma	Unknown	PT2N0
CRC-43	Colon	Adenocarcinoma	Polypoid	pT3N0
CRC-44	Rectum	Adenocarcinoma	Unknown	pT3N
CRC-45	Rectum	Adenocarcinoma	Ulcerative	pT3N1a
CRC-46	Colon	Adenocarcinoma	Ulcerative	pT3N1bM1
CRC-47	Colon	Adenocarcinoma	Ulcerative	pT3N0
CRC-48	Colon	Adenocarcinoma	Unknown	pT3N1b
CRC-49	Colon	Adenocarcinoma	Polypoid	pT3N0
CRC-50	Colon	Mucinous adenocarcinoma	Ulcerative	pT3N1
CRC-51	Colon	Adenocarcinoma	Unknown	pT3N1b
CRC-52	Colon	Adenocarcinoma	Polypoid	PT1N0
CRC-53	Rectum	Adenocarcinoma	Polypoid	pT3N0
CRC-54	Rectum	Adenocarcinoma	Polypoid	pT3N0
CRC-55	Colon	Adenocarcinoma	Unknown	pT3N0
CRC-56	Rectum	Adenocarcinoma	Ulcerative	pT4aN1b
CRC-57	Colon	Adenocarcinoma	Ulcerative	pT3N1c
CRC-58	Rectum	Mucinous adenocarcinoma	Polypoid	pT3N2b
CRC-59	Rectum	Adenocarcinoma	Unknown	pT3N0
CRC-60	Colon	Adenocarcinoma	Ulcerative	pT4aN1a
CRC-61	Rectum	Adenocarcinoma	Polypoid	PT2N0
CRC-62	Rectum	Adenocarcinoma	Unknown	pT3N1a
CRC-63	Colon	Adenocarcinoma	Ulcerative	pT3N0
CRC-64	Rectum	Adenocarcinoma	Ulcerative	pT3N0
CRC-65	Rectum	Adenocarcinoma	Ulcerative	pT3N2a
CRC-66	Rectum	Adenocarcinoma	Ulcerative	PT2N0
CRC-67	Ileocecus	Adenocarcinoma	Ulcerative	pT4aN1b
CRC-68	Colon	Adenocarcinoma	Polypoid	pT3N1c
CRC-69	Colon	Adenocarcinoma	Unknown	pT3N1b
CRC-70	Rectum	Adenocarcinoma	Polypoid	PT2N0
CRC-71	Rectum	Adenocarcinoma	Ulcerative	pT3N0
CRC-72	Rectum	Adenocarcinoma	Unknown	pT3N1b
CRC-73	Colon	Adenocarcinoma	Ulcerative	pT3N0
CRC-74	Colon	Adenocarcinoma	Polypoid	T4aN0
CRC-75	Rectum	Adenocarcinoma	Ulcerative	pT3N1a
CRC-76	Rectum	Adenocarcinoma	Unknown	pT3N1a
CRC-77	Rectum	Adenocarcinoma	Ulcerative	pT3N0
CRC-78	Rectum	Adenocarcinoma	Ulcerative	pT3N0
CRC-79	Colon	Mucinous adenocarcinoma	Polypoid	pT3N0
CRC-80	Rectum	Adenocarcinoma	Ulcerative	pT3N2b
CRC-81	Colon	Adenocarcinoma	Unknown	pT3N1
CRC-82	Rectum	Adenocarcinoma	Unknown	pT3N0
CRC-83	Rectum	Adenocarcinoma	Polypoid	pT3N0
CRC-84	Rectum	Adenocarcinoma	Ulcerative	pT3N0
CRC-85	Rectum	Adenocarcinoma	Ulcerative	pT3N0
CRC-86	Rectum	Mucinous adenocarcinoma	Ulcerative	pT3N1b
CRC-87	Colon	Adenocarcinoma	Ulcerative	pT3N0
CRC-88	Colon	Adenocarcinoma	Unknown	pT3N1b
CRC-89	Rectum	Adenocarcinoma	Polypoid	pT3N1c
CRC-90	Colon	Adenocarcinoma	Ulcerative	PT2N0
CRC-91	Colon	Mucinous adenocarcinoma	Polypoid	pT3N1b
CRC-92	Colon	Mucinous adenocarcinoma	Polypoid	pT3N0
CRC-93	Rectum	Adenocarcinoma	Polypoid	pT3N0
CRC-94	Colon	Adenocarcinoma	Ulcerative	pT3N0
CRC-95	Rectum	Adenocarcinoma	Ulcerative	pT3N1b
CRC-96	Colon	Adenocarcinoma	Polypoid	pT4aN1a
CRC-97	Rectum	Adenocarcinoma	Ulcerative	pT4bN1b

CRC-98	Colon	Adenocarcinoma	Ulcerative	pT3N2a
CRC-99	Colon	Adenocarcinoma	Polypoid	pT3N0
CRC-100	Female	Adenocarcinoma	Polypoid	pT3N0

Recruitment

Human blood samples were obtained from patients with colon adenocarcinoma under the approval of the Institutional Review Board of the China-Japan Friendship Hospital.

Ethics oversight

All procedures were approved by the Institutional Review Board of the China-Japan Friendship Hospital (No. 2019-50-Q07), and the informed consent was obtained from all subjects (in accordance with the Helsinki Declaration).

Note that full information on the approval of the study protocol must also be provided in the manuscript.

Clinical data

Policy information about clinical studies

All manuscripts should comply with the ICMJEguidelines for publication of clinical research and a completedCONSORT checklist must be included with all submissions.

Clinical trial registration

Provide the trial registration number from ClinicalTrials.gov or an equivalent agency.

Study protocol

Note where the full trial protocol can be accessed OR if not available, explain why.

Data collection

Describe the settings and locales of data collection, noting the time periods of recruitment and data collection.

Outcomes

Describe how you pre-defined primary and secondary outcome measures and how you assessed these measures.

Dual use research of concern

Policy information about dual use research of concern

Hazards

Could the accidental, deliberate or reckless misuse of agents or technologies generated in the work, or the application of information presented in the manuscript, pose a threat to:

No	Yes	
☐	☐	Public health
☐	☐	National security
☐	☐	Crops and/or livestock
☐	☐	Ecosystems
☐	☐	Any other significant area

Experiments of concern

Does the work involve any of these experiments of concern:

No	Yes	
☐	☐	Demonstrate how to render a vaccine ineffective
☐	☐	Confer resistance to therapeutically useful antibiotics or antiviral agents
☐	☐	Enhance the virulence of a pathogen or render a nonpathogen virulent
☐	☐	Increase transmissibility of a pathogen
☐	☐	Alter the host range of a pathogen
☐	☐	Enable evasion of diagnostic/detection modalities
☐	☐	Enable the weaponization of a biological agent or toxin
☐	☐	Any other potentially harmful combination of experiments and agents

ChIP-seq

Data deposition

☐ Confirm that both raw and final processed data have been deposited in a public database such as GEO.

☐ Confirm that you have deposited or provided access to graph files (e.g. BED files) for the called peaks.

Data access links
May remain private before publication.

For "Initial submission" or "Revised version" documents, provide reviewer access links. For your "Final submission" document, provide a link to the deposited data.

Files in database submission	*Provide a list of all files available in the database submission.*
Genome browser session (e.g. UCSC)	*Provide a link to an anonymized genome browser session for "Initial submission" and "Revised version" documents only, to enable peer review. Write "no longer applicable" for "Final submission" documents.*

Methodology

Replicates	*Describe the experimental replicates, specifying number, type and replicate agreement.*
Sequencing depth	*Describe the sequencing depth for each experiment, providing the total number of reads, uniquely mapped reads, length of reads and whether they were paired- or single-end.*
Antibodies	*Describe the antibodies used for the ChIP-seq experiments; as applicable, provide supplier name, catalog number, clone name, and lot number.*
Peak calling parameters	*Specify the command line program and parameters used for read mapping and peak calling, including the ChIP, control and index files used.*
Data quality	*Describe the methods used to ensure data quality in full detail, including how many peaks are at FDR 5% and above 5-fold enrichment.*
Software	*Describe the software used to collect and analyze the ChIP-seq data. For custom code that has been deposited into a community repository, provide accession details.*

Flow Cytometry

Plots

Confirm that:

- [x] The axis labels state the marker and fluorochrome used (e.g. CD4-FITC).
- [x] The axis scales are clearly visible. Include numbers along axes only for bottom left plot of group (a 'group' is an analysis of identical markers).
- [x] All plots are contour plots with outliers or pseudocolor plots.
- [x] A numerical value for number of cells or percentage (with statistics) is provided.

Methodology

Sample preparation	To detect viral replication, HEK293T, MEFs and iBMDM were infected with VSV-GFP, and GFP+ cells were measured by flow cytometry.<br>Lymphocytes from lymph node, spleen and colon were isolated and incubated with specific antibodies for 30 min at room temperature.
Instrument	Flow cytometry analysis: FACSVerse, BD Biosciences.
Software	Flow cytometry data was collected using the FACSuite Software Bundle v1.0 (BD Biosciences). Flow cytometry data was analyzed using the FlowJo v7.6.1 software.
Cell population abundance	Murine immune cells from colon were enriched using percoll reagent and purity of sorted cells was > 90% (determined by flow cytometry).
Gating strategy	Live lymphocytes were identified by cell size and granularity in a FSC/SSC plot. Gates indicating boundaries between positive and negative were based on the isotype control staining. Expression of indicated proteins were evaluated on these populations as indicated in the figures and figure legends.

- [x] Tick this box to confirm that a figure exemplifying the gating strategy is provided in the Supplementary Information.

Magnetic resonance imaging

Experimental design

Design type	*Indicate task or resting state; event-related or block design.*
Design specifications	*Specify the number of blocks, trials or experimental units per session and/or subject, and specify the length of each trial or block (if trials are blocked) and interval between trials.*
Behavioral performance measures	*State number and/or type of variables recorded (e.g. correct button press, response time) and what statistics were used to establish that the subjects were performing the task as expected (e.g. mean, range, and/or standard deviation across subjects).*

Acquisition

Imaging type(s) | *Specify: functional, structural, diffusion, perfusion.*

Field strength | *Specify in Tesla*

Sequence & imaging parameters | *Specify the pulse sequence type (gradient echo, spin echo, etc.), imaging type (EPI, spiral, etc.), field of view, matrix size, slice thickness, orientation and TE/TR/flip angle.*

Area of acquisition | *State whether a whole brain scan was used OR define the area of acquisition, describing how the region was determined.*

Diffusion MRI ☐ Used ☐ Not used

Preprocessing

Preprocessing software | *Provide detail on software version and revision number and on specific parameters (model/functions, brain extraction, segmentation, smoothing kernel size, etc.).*

Normalization | *If data were normalized/standardized, describe the approach(es): specify linear or non-linear and define image types used for transformation OR indicate that data were not normalized and explain rationale for lack of normalization.*

Normalization template | *Describe the template used for normalization/transformation, specifying subject space or group standardized space (e.g. original Talairach, MNI305, ICBM152) OR indicate that the data were not normalized.*

Noise and artifact removal | *Describe your procedure(s) for artifact and structured noise removal, specifying motion parameters, tissue signals and physiological signals (heart rate, respiration).*

Volume censoring | *Define your software and/or method and criteria for volume censoring, and state the extent of such censoring.*

Statistical modeling & inference

Model type and settings | *Specify type (mass univariate, multivariate, RSA, predictive, etc.) and describe essential details of the model at the first and second levels (e.g. fixed, random or mixed effects; drift or auto-correlation).*

Effect(s) tested | *Define precise effect in terms of the task or stimulus conditions instead of psychological concepts and indicate whether ANOVA or factorial designs were used.*

Specify type of analysis: ☐ Whole brain ☐ ROI-based ☐ Both

Statistic type for inference (See Eklund et al. 2016) | *Specify voxel-wise or cluster-wise and report all relevant parameters for cluster-wise methods.*

Correction | *Describe the type of correction and how it is obtained for multiple comparisons (e.g. FWE, FDR, permutation or Monte Carlo).*

Models & analysis

n/a	Involved in the study
☐	☐ Functional and/or effective connectivity
☐	☐ Graph analysis
☐	☐ Multivariate modeling or predictive analysis

Functional and/or effective connectivity | *Report the measures of dependence used and the model details (e.g. Pearson correlation, partial correlation, mutual information).*

Graph analysis | *Report the dependent variable and connectivity measure, specifying weighted graph or binarized graph, subject- or group-level, and the global and/or node summaries used (e.g. clustering coefficient, efficiency, etc.).*

Multivariate modeling and predictive analysis | *Specify independent variables, features extraction and dimension reduction, model, training and evaluation metrics.*
